# Utilizing predictive machine-learning modelling unveils feature-based risk assessment system for hyperinflammatory patterns and infectious outcomes in polytrauma

**DOI:** 10.3389/fimmu.2023.1281674

**Published:** 2023-12-12

**Authors:** Melanie Fachet, Raghava Vinaykanth Mushunuri, Christian B. Bergmann, Ingo Marzi, Christoph Hoeschen, Borna Relja

**Affiliations:** ^1^ Institute for Medical Technology, Medical Systems Technology, Faculty of Electrical Engineering and Information Technology, Otto von Guericke University Magdeburg, Magdeburg, Germany; ^2^ Translational and Experimental Trauma Research, Department of Trauma, Hand, Plastic and Reconstructive Surgery, Ulm University Medical Center, University Ulm, Ulm, Germany; ^3^ Department of Trauma, Hand and Reconstructive Surgery, Medical Faculty, Goethe University Frankfurt, Frankfurt, Germany

**Keywords:** risk assessment, clinical decision making, classification, explainability, SHAP values, blood, biomarker

## Abstract

**Purpose:**

Earlier research has identified several potentially predictive features including biomarkers associated with trauma, which can be used to assess the risk for harmful outcomes of polytraumatized patients. These features encompass various aspects such as the nature and severity of the injury, accompanying health conditions, immune and inflammatory markers, and blood parameters linked to organ functioning, however their applicability is limited. Numerous indicators relevant to the patients` outcome are routinely gathered in the intensive care unit (ICU) and recorded in electronic medical records, rendering them suitable predictors for risk assessment of polytraumatized patients.

**Methods:**

317 polytraumatized patients were included, and the influence of 29 clinical and biological features on the complication patterns for systemic inflammatory response syndrome (SIRS), pneumonia and sepsis were analyzed with a machine learning workflow including clustering, classification and explainability using SHapley Additive exPlanations (SHAP) values. The predictive ability of the analyzed features within three days after admission to the hospital were compared based on patient-specific outcomes using receiver-operating characteristics.

**Results:**

A correlation and clustering analysis revealed that distinct patterns of injury and biomarker patterns were observed for the major complication classes. A k-means clustering suggested four different clusters based on the major complications SIRS, pneumonia and sepsis as well as a patient subgroup that developed no complications. For classification of the outcome groups with no complications, pneumonia and sepsis based on boosting ensemble classification, 90% were correctly classified as low-risk group (no complications). For the high-risk groups associated with development of pneumonia and sepsis, 80% of the patients were correctly identified. The explainability analysis with SHAP values identified the top-ranking features that had the largest impact on the development of adverse outcome patterns. For both investigated risk scenarios (infectious complications and long ICU stay) the most important features are SOFA score, Glasgow Coma Scale, lactate, GGT and hemoglobin blood concentration.

**Conclusion:**

The machine learning-based identification of prognostic feature patterns in patients with traumatic injuries may improve tailoring personalized treatment modalities to mitigate the adverse outcomes in high-risk patient clusters.

## 1 Introduction

Severe trauma stands as a noteworthy global public health challenge, constituting nearly 8% of all deaths and resulting in over 4.4 million deaths worldwide annually (1). The World Health Organization highlights road traffic accidents, suicides and homicides as primary contributors to injury and violence-related deaths (1). Advances in prehospital transport and resuscitation strategies have significantly influenced the patterns of traumatic deaths over recent decades (2). Trauma-related fatalities display a bimodal distribution, with a majority occurring in the initial days following the injury, often due to severe head injury or uncontrollable bleeding. Survivors of the initial traumatic event frequently confront a critical illness characterized by prolonged stays in intensive care units (ICU) or hospitals (length of stays, LOS), along with an elevated risk of inflammatory complications (3, 4).

In the realm of post-injury scenarios, the pivotal determinant of outcomes lies in the concurrent immuno-inflammatory response (5, 6). When this response is appropriately controlled in terms of both intensity and duration, it plays a crucial role in restoring the host homeostasis. Conversely, an irregular response is associated with the onset of multiple organ dysfunction syndrome (MODS), culminating in prolonged critical illness and a sustained elevated susceptibility to complications and mortality even post-discharge (7). Experiencing multiple traumas triggers substantial blood loss and the accumulation of necrotic or devitalized tissue within an ischemic-hypoxic environment devoid of oxygen and nutrients, both of which contribute to coagulatory and inflammatory alterations. The inflammatory response following polytrauma plays a pivotal role in the body`s molecular defense mechanisms. The initial phase of inflammation after polytrauma involves two coordinated processes: the systemic inflammatory response syndrome (SIRS) representing a pro-inflammatory reaction, and the compensatory anti-inflammatory response syndrome constituting an anti-inflammatory reaction (8). SIRS manifests through changes in heart rate, respiratory rate, temperature regulation, and activation of immune cells. In the typical course of the inflammatory response following trauma, a delicate equilibrium is maintained between the pro- and anti-inflammatory reactions, ensuring biological homeostasis and fostering controlled regeneration processes that support a normal recovery without significant complications. However, an exaggerated inflammatory response following trauma has the potential to simultaneously activate both innate pro- and anti-inflammatory mediators while suppressing adaptive immunity, that can lead to the early onset of multiple organ dysfunction syndrome (MODS) (9). Furthermore, an extended and dysregulated immune-inflammatory state is linked to delayed recovery and complications, particularly the emergence of late-stage MODS. The intricate interplay of these factors can result in severe SIRS, acute respiratory distress syndrome, sepsis, acute kidney injury, and ultimately MODS. Various influencing factors include the type of injured tissue, post-injury surgical management, age, sex, genetics, and critically, underlying comorbidities and physical conditions, encompassing both exogenous and endogenous factors (7, 10). Accurately identifying the risks faced by patients in ICUs after traumatic injuries is essential for tailoring existing treatment strategies and mitigating subsequent complications, particularly in high-risk patient sub-groups. Consequently, the identification of patients with a heightened risk of unfavorable outcomes can aid clinicians in ascertaining the optimal care setting and treatment modalities during trauma management (11).

Within clinical medicine, there is an increasing interest towards utilizing model predictions. Machine learning tools have been applied to predict outcomes such as acute kidney injury or sepsis and septic shock in hospitalized patients (12–16). Nevertheless, there exists a scarcity of studies exploring the use of distinct machine learning algorithms for predicting risks related to infectious complications and LOS in polytraumatized patients. To address this gap in knowledge, we conducted a cohort study to thoroughly assess the performance of various machine learning algorithms in identifying features including biomarkers for risk assessment and informing clinical decision-making. The aim of this study is to create a prognostic machine learning approach that combines data from electronic medical records, including patient demographics, injury patterns and severity, and laboratory data of polytraumatized patients. The methodology outlined in this study involves the sequential application of feature selection, correlation analyses, clustering, classification, and explainability techniques to anticipate adverse outcome patterns in a cohort of polytrauma patients. Serial blood measurements taken within the first three days of hospital admission, along with routinely recorded data from electronic medical records, were utilized. The risk classification model, employing an ensemble classification algorithm, demonstrated accurate predictions of the risk of infectious complications and prolonged stays in the ICU or hospital with high precision when tested on an independent patient dataset. Furthermore, critical clinical and inflammatory biomarkers for the early-stage prediction of risk patterns following hospitalization were identified.

## 2 Materials and methods

### 2.1 Data collection and sampling

A total of 317 polytraumatized patients were enrolled in the study at the emergency department (ED) of the University Hospital Frankfurt of the Goethe University from 2012 to 2016. The enrollment was done prospectively in accordance with the ethical committee approval and the Declaration of Helsinki as well as following the Strengthening the Reporting of Observational Studies in Epidemiology guidelines (17). All included patients provided the written informed consent forms themselves or informed consent was obtained from the nominated legally authorized representative consented on the behalf of participants as approved by the ethical committee (312/10). Part of the data that were obtained in the ED was published before (18). The study included polytraumatized patients with an injury severity score (ISS) of 16 or higher, aged 18 or above, who were admitted to the ICU and expected to survive beyond the initial 24 h post-injury. Certain exclusion criteria were applied, such as known pre-existing immunological disorders, immunosuppressive and anti-coagulant medication, burns, concomitant acute myocardial infarction, or thromboembolic events. All patients were treated in the ED according to the Advanced Trauma Life Support standards and the guidelines for polytrauma management (19).

Demographic and clinical data were collected from electronic medical records, including age, sex, ISS, abbreviated injury scale (AIS), length of stay (LOS) in the ICU and hospital LOS. Various severity scores were calculated daily during hospitalization, including the Glasgow Coma Scale (GCS), acute physiology and chronic health evaluation (APACHE) II and sequential organ failure assessment (SOFA) score.

Blood samples were collected daily on ten consecutive days post-injury from the patients in pre-chilled ethylenediaminetetraacetic acid tubes (BD vacutainer, Becton Dickinson Diagnostics, Aalst, Belgium) and kept on ice. Blood was centrifuged at 2000×g for 15 min at 4°C and the supernatant was stored at -80°C until IL-6 or IL-10 analyses according to the manufacturer`s instructions (IL-6 and IL-10 Elipair ELISA-Assay Diaclone, Hoelzel Diagnostica, Cologne, Germany). Blood was withdrawn daily as follows: the initial blood draw upon arrival in the ED; within 24h (D1), 48h (D2) and 72h (D3) of admission to the ED as a part of routine care. Data were obtained from ED to day ten post-injury. For the machine-learning approach described in Section 2.2, the mean of the results from D1-D3 were applied for analysis to predict the trauma associated outcome patterns at an early stage after admission to the ED. In some instances, it was not possible to obtain samples due to conflicts with clinical care or removal from the ICU.

#### 2.1.1 Optimization of the study cohort

The polytrauma cohort study was designed to investigate complications such as SIRS, pneumonia, and sepsis in trauma patients. The prospectively collected data were based on previous theory on risk factors for polytrauma such as injury patterns, injury severity scores, blood markers for organ dysfunction as well as immune and inflammatory markers. For the proposed classification model, a subset of all collected data was selected as candidate predictor variables based on the consideration that data should be readily available and routinely collected also in the ICU. We considered 29 variables as candidate predictors and two variables (procalcitonin and antithrombin activity) were excluded due to missing values exceeding 70%.

#### 2.1.2 Outcomes

The primary outcome of the predictive model was the classification of polytrauma patients into different complication patterns: no complications (n=194), SIRS (n=51), pneumonia (n=39) and sepsis (n=33). The criteria for diagnosing SIRS, pneumonia, and sepsis were based on established definitions and guidelines. Briefly, SIRS definition met the following criteria: heart rate >90 beats per minute; respiratory rate >20 breaths per minute or arterial carbon dioxide tension <32 mmHg; body temperature >38°C or <36°C; and white blood cell count >12.000 cells/mm^3^ or <4.000 cells/mm^3^, or with >10% immature (band) forms. SIRS was diagnosed when two or more of these criteria were fulfilled. The diagnose of pneumonia was defined by clinical, radiologic, and bacteriologic findings including new pulmonary infiltrates on chest X-ray as well as one of the following criteria: positive blood culture, bronchial alveolar lavage, and/or sputum culture (20). Sepsis was assessed by applying the 2005 criteria outlined by the International Sepsis Forum diagnosing sepsis by fulfilling SIRS criteria and having a proven infection (21). Apart from the inflammatory complication patterns described above, we have defined a second outcome scenario associated with a long stay in the ICU or total-hospital stay. In order to identify and compare biomarker risk patterns corresponding to a long LOS in LCU or hospital, we have defined a threshold of ICU stay for >14 days and/or LOS for >30 days as a separate class for polytrauma patients at higher risk.

### 2.2 Model development

#### 2.2.1 Feature extraction and correlation analysis

The feature extraction and correlation analysis were performed based on the Spearman’s correlation coefficient with respect to their importance on the outcome class, and features with positive correlation and which has significant correlation with class labels were selected for the classification workflow described in Sec. 2.3.

#### 2.2.2 Filtered *k*-means clustering

To identify distinct patient risk patterns, present in the first 3 days after injury, the patients outcome risk class for the 317 patients admitted to the ICU after trauma were subjected to filtered k-means clustering. The euclidian distance was used as a distance function with S=15 as seed number.

### 2.3 Classification

The classification was carried out using Python and pycharm. Missing data was imputed using k-nearest-neighbor imputation with the number of neighbors n=3. Hyperparameter of different classifiers (random forest, naive bayes and ensemble classifiers) were optimized to minimize the final prediction error.

#### 2.3.1 Synthetic data creation

The original data set contained information on 317 patients, with 194 patients having no complications and 123 patients exhibiting hyperinflammatory or infectious complication patterns, such as SIRS (n=51), pneumonia (n=39) and sepsis (n=33). The class imbalance in the data set for the adverse outcomes (pneumonia and sepsis as infectious complications or risk for long ICU stay or long total hospitalization time) was addressed using Synthetic Minority Oversampling Technique (SMOTE), which generates synthetic training examples by linear interpolation for the minority class (22). These synthetic training examples are generated by randomly selecting one or more of the k-nearest neighbors for each example in the minority class. SMOTE technique is applied for oversampling in which each sampled instance of minority class is generated using 3 nearest neighbors. After the oversampling process, the data is reconstructed, and various classification techniques can be employed for the processed data.

#### 2.3.2 Evaluation of model performance

The model performance evaluation was done by a k-fold cross validation ranging from 2-fold to 20-fold during the model development using a train-test split for the patient data. In this method, 20% of the patients were excluded while training the model and the excluded patients were then used to test the model. The model performance was assessed using area under the receiver operating characteristic (ROC) curve (AUC), F1 score and predication accuracy. The best performing model from the above criteria is then used to interpret the biomarker importance with exploitability methods.

#### 2.3.3 Model interpretation using SHAP values

We have explained our models that are used for classification using SHAP (SHapley Additive exPlanations) values that offer a high level of interpretability for our proposed risk analysis model (23). The SHAP values for each patient feature explain the intensity and direction of impact on predicting the class labels (11). The SHAP tree explainer was used to explain the XGBoost prediction, which uses decision trees for classification, and to visualize the results in beeswarm plots.

### 2.4 Statistical analysis

Descriptive statistics and non-parametric tests were used to analyze the demographic, clinical, and inflammatory data using Origin (Version 2019b). Descriptive measures included mean, median, standard error of the mean, and interquartile range for continuous variables and percentages for categorical variables. Furthermore, non-parametric tests were used for testing whether group means, or medians are distributed the same across groups by ranking each attribute from our data set. The non-parametric Wilcoxon rank-sum test of the null hypothesis between two independent samples (patient subgroup showing no complications with patient subgroups showing complications) was tested in MATLAB using the function rank-sum. The extension of the Wilcoxon rank-sum test for more than two groups, the Kruskal-Wallis ANOVA test was conducted in Origin (Version 2019b). A significant Kruskal–Wallis test indicates that at least one sample stochastically dominates one other sample.

## 3 Results

### 3.1 Demographics and outcome-related features for risk assessment

**Figure 1:**
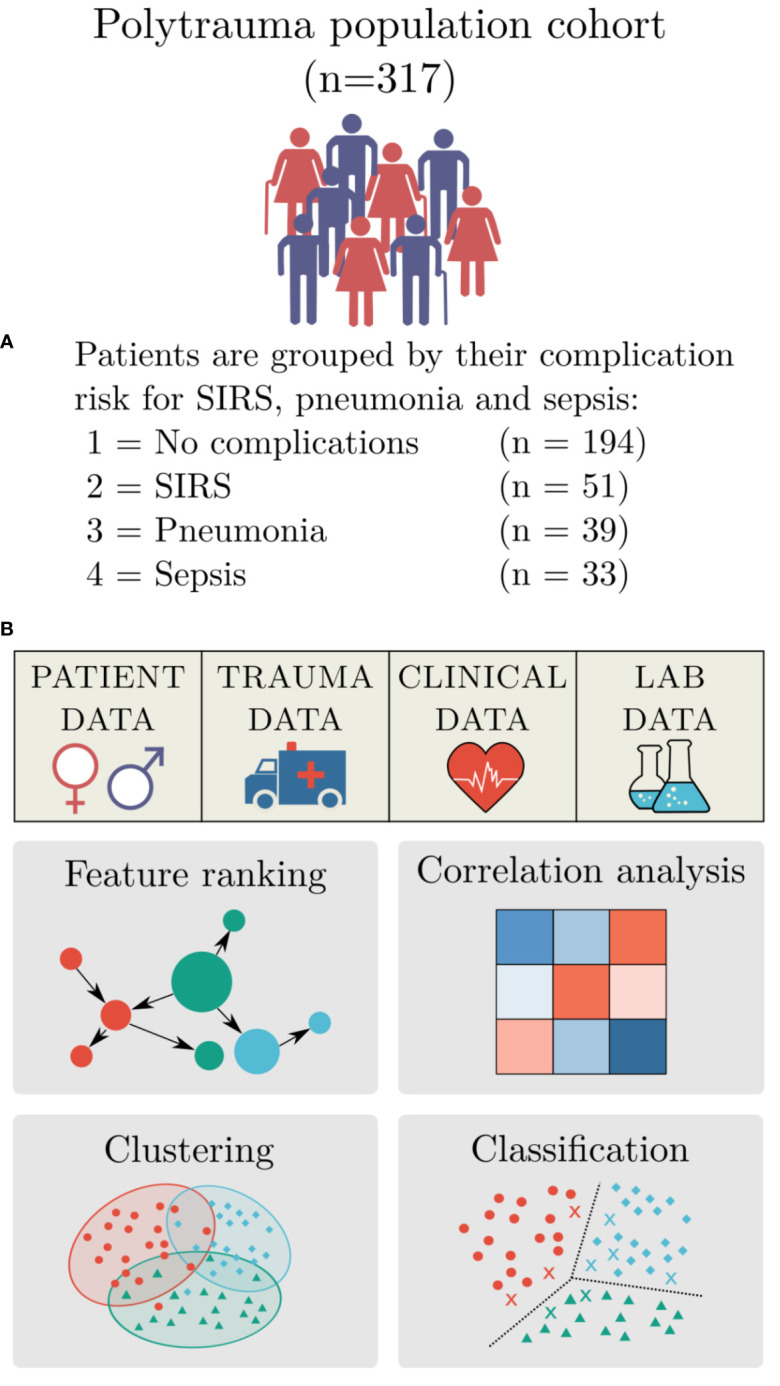
Study design. **(A)** Summary of the polytrauma patient cohort, including their outcome pattern: group 1 - no complications (n=194), group 2 – systemic inflammatory response syndrome (SIRS) (n=51), group 3 - pneumonia (n=39), and group 4 - sepsis (n=33). **(B)** Study design for machine-learning-based data analysis for risk assessment included: Identification of feature importance, correlation analysis, clustering and classification.

The aim of this study was to utilize feature selection and correlation analysis to identify the most important features from clinical patients` data for polytrauma outcome classification. For optimization of our study cohort, patients discharged prior to day 10 from the ICU and 21 patients who died before discharge were excluded, which yielded a total of 317 patients used in the current study. We utilized feature extraction, clustering, and classification to identify attributes correlating with complication patterns (SIRS, pneumonia, and sepsis) observed in the patient cohort (**Figure 1**). Demographic metrics, injury patterns and other outcome related characteristics for the 317 trauma patients for the analysis are shown in **Table 1** and **Supplementary Figures S1**–**S4**.

**Table 1:** Overview of the polytrauma patient cohort and associated injury patterns.

	No complications (n=194)	SIRS (n=51)	Pneumonia (n=39)	Sepsis (n=33)	p < 0.05
Demographics					
Age (y)	45.9 ± 1.3	44.7 ± 2.4	53.5 ± 3.0	45.2 ± 3.2	n.s.
Sex (m/f)	149/45	43/8	33/6	24/9	n.s.
ISS	24.8 ± 0.7	27.6 ± 1.3	26.7 ± 1.7	32.7 ± 1.8	a, b
AIS					
Head	2.1 ± 0.1	2.3 ± 0.2	3.4 ± 0.3	1.7 ± 0.3	b, c, d,
Thorax	2.0 ± 1.1	2.3 ± 0.2	1.5 ± 0.3	2.7 ± 0.3	b
Abdomen	0.8 ± 0.1	0.5 ± 0.1	0.7 ± 0.2	1.0 ± 0.3	n.s.
Extremities	1.1 ± 0.1	1.4 ± 0.2	0.8 ± 0.2	1.8 ± 0.2	a, b
Cytokines					
IL-6 ED (pg/ml)	153.5 ± 21.4	180.6 ± 50.8	266.3 ± 78.3	343.5 ± 118.4	n.s.
IL-6 D1 (pg/ml)	189.8 ± 21.3	264.7 ± 69.8	269.3 ± 53.5	329.5 ± 83.8	n.s.
IL-6 D2 (pg/ml)	158.3 ± 34.8	112.0 ± 20.8	175.5 ± 47.4	304.3 ± 86.0	n.s.
IL-6 D3 (pg/ml)	69.7 ± 8.8	56.9 ± 8.3	105.0 ± 19.8	146.7 ± 32.0	a, c
IL-10 ED (pg/ml)	90.4 ± 12.6	108.2 ± 26.1	146.2 ± 38.6	138.2 ± 31.7	n.s.
IL-10 D1 (pg/ml)	15.6 ± 2.5	20.1 ± 5.1	34.8 ± 9.1	51.0 ± 15.1	a, c
IL-10 D2 (pg/ml)	10.0 ± 1.9	9.2 ± 3.0	7.0 ± 1.3	18.6 ± 8.5	n.s.
IL-10 D3 (pg/ml)	6.6 ± 1.8	10.7 ± 3.5	6.1 ± 1.2	33.4 ± 18.6	n.s.
Outcome					
SIRS ED (n, %)	7, 3.6	29, 56.9	10, 25.6	14, 42.4	d, e
SIRS D1 (n, %)	0, 0	20, 39.2	11, 28.2	15, 45.4	e
SIRS D2 (n, %)	0, 0	19, 37.3	7, 17.9	9, 27.3	d, e
SIRS D3 (n, %)	0, 0	17, 33.3	8, 20.5	15, 45.4	b, e
SIRS ED-D10 (n, %)	7, 3.6	51, 100	16, 41.0	33, 100	b, d, e
SIRS total (d)	0.1 ± 0.1	2.8 ± 0.3	2.0 ± 0.5	7.5 ± 1.0	b, d, e
MV (d)	2.9 ± 0.3	6.6 ± 0.6	7.7 ± 0.7	2.9 ± 4.0	e
ICU LOS (d)	6.6 ± 0.5	12.4 ± 1.3	12.6 ± 1.5	7.8 ± 0.7	e
Hospital LOS (d)	16.4 ± 0.8	25.5 ± 2.9	19.6 ± 1.9	36.6 ± 4.2	f, g
In-hospital mortality (n, %)	22, 11.3	4, 7.8	4, 10.3	1, 3.0	n.s.

D, day; d, days; ED, emergency department; f, female; ICU, intensive care unit; ISS, injury severity score; IL, interleukin; LOS, length of stay; y, years; m, male; ml, milliliter; MV, mechanical ventilation; n, sample size; n.s., not significant; pg, picogram; SIRS, systemic inflammatory response syndrome. Significant differences (p <0.05) between the groups are indicated as follows: a: no complications vs. sepsis, b: pneumonia vs. sepsis, c: no complications vs. pneumonia, d: SIRS vs. pneumonia, e: no complications vs. all, f: no complications vs. SIRS, g: sepsis vs. all.

Subsequently, the patient cohort (n=317) was divided into four sub-groups based on commonly occurring complications of trauma patients. Out of 317 patients, 16.1% developed SIRS, 12.3% had pneumonia and 10.5% were septic within 10 days after admission to the ED, whereas the majority of 61.2% patients showed none of the previously mentioned complications. The patient sub-group with sepsis had the longest hospital stay (**Table 1**). No statistically significant differences among the sub-groups in regard to age or sex were detected. The sub-group with sepsis had significantly higher ISS compared to the no complication and pneumonia sub-groups (**Table 1**).

### 3.2 SIRS patients showed the weakest feature correlation among the investigated complications

**Figure 2:**
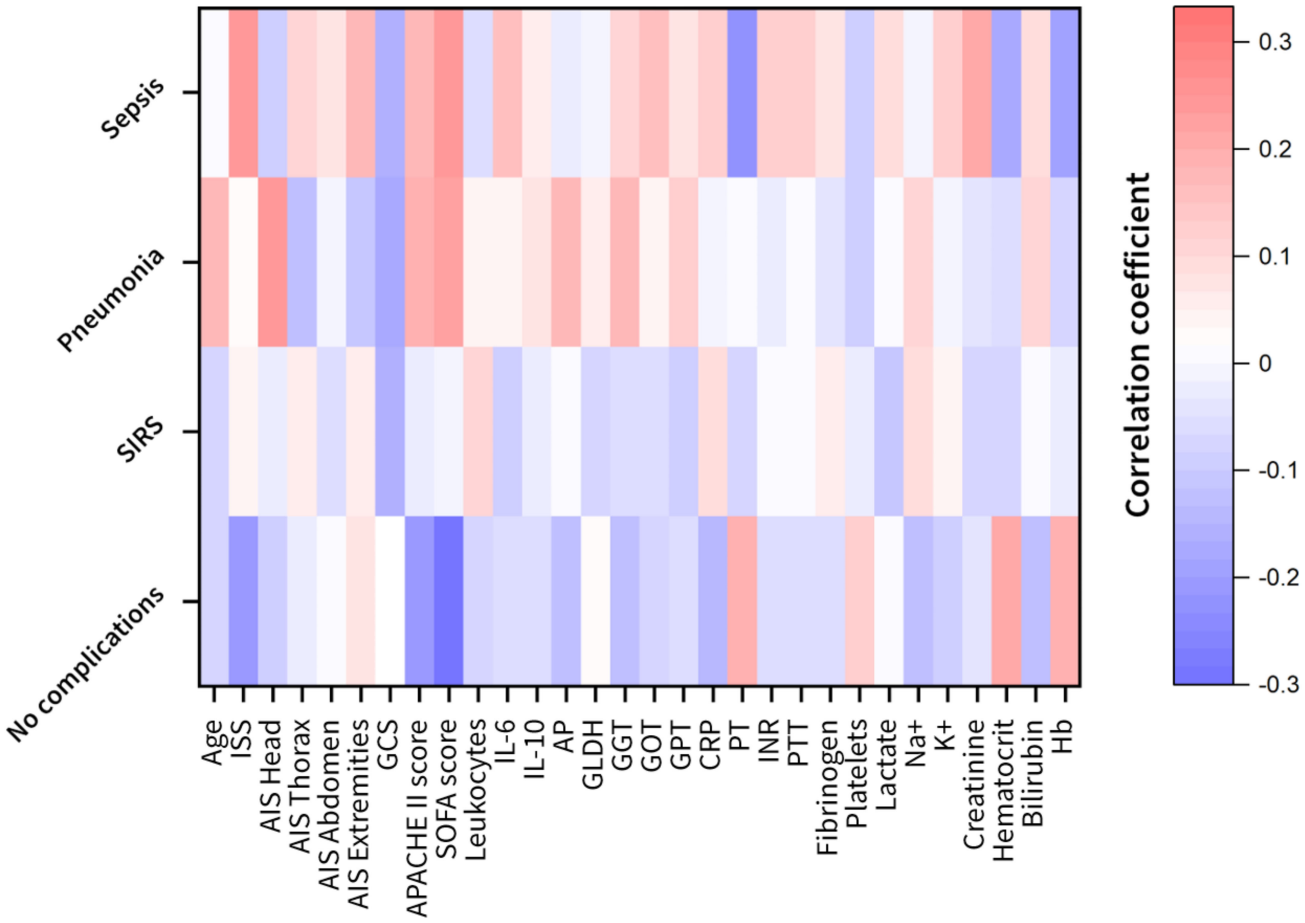
Heat maps of the feature correlation of the clinical attributes with respect to the different complication classes (no complication, systemic inflammatory response syndrome (SIRS), pneumonia and sepsis). Correlation strength is color-coded (red to white: positive correlations; white to blue: negative correlations). AIS, abbreviated injury scale; AP, alkaline phosphatase; APACHE, acute physiology and chronic health evaluation; CRP, C-reactive protein; GCS, glasgow coma scale; GLDH, glutamate dehydrogenase; GGT, gamma glutamyl transpeptidase; GOT, glutamic oxaloacetic transaminase; GPT, glutamic pyruvic transaminase; Hb, hemoglobin; IL, interleukin; INR, international normalized ratio; ISS, injury severity score; K, potassium; Na, sodium; PT, prothrombin time; PTT, partial thromboplastin time; SOFA, sequential organ failure assessment.

The prospectively collected data was subjected to the non-parametric Wilcoxon rank-sum test for two independent samples (patient sub-group showing no complications with patient-subgroups showing complications) and the Kruskal-Wallis ANOVA test for multiple independent samples with a significance level of p<0.05. The results in **Table 2** show that the least statistical significance is observed for the SIRS group with 8 out of 29 features that reject the null hypothesis, followed by the pneumonia group with 9 out of 29 features (**Table 2**). The highest degree of significance is observed for the septic group with 19 out of 29 attributes. The Glasgow Coma Scale based on motor responsiveness, verbal performance and eye opening to an appropriate stimulus was designed to assess the depth and duration coma and impaired consciousness after traumatic injuries is showing a high significance in all complication sub-groups compared to the reference group having no complications (**Table 2**). Moreover, the partial thromboplastin time (PTT) that is a measure for the overall clotting speed of the blood by the intrinsic pathway and common pathway of coagulation as well as hemoglobin (Hb), the oxygen-binding metalloprotein of erythrocytes were found to be significant in all risk groups (**Table 2**). Based on the feature importance ranking and the correlation analysis, the most informative features in classifying a certain complication pattern are shown in **Figure 2** and **Supplementary Figure S5**. The highest ranking and therefore most-informative features for infectious complications are the SOFA score (rank 1) and the GCS (rank 3, **Supplementary Figure S5**). Besides, the overall injury severity represented by the ISS (rank 6) has shown that the traumatic injury pattern is of relevance for the risk assessment with AIS Head (rank 11) among the top 20 ranking features. The Hb level as a measure for assessing acute blood loss after trauma were ranked on position 13, respectively. The transpeptidase and transaminases gamma glutamyl transpeptidase (GGT, rank 5), glutamic oxaloacetic transaminase (GOT, rank 17) and glutamic pyruvic transaminase (GPT, rank 18) were found to be of high relevance for identification of adverse outcome patterns in the investigated patient cohort (**Supplementary Figure S5**).

**Table 2:** Kruskal-Wallis ANOVA test and Wilcoxon rank sum test for discrimination between patient sub-groups with no complications with patient sub-groups having systemic inflammatory response syndrome (SIRS), pneumonia or sepsis for a significance level of p <0.05.

Attribute	Kruskal-Wallis ANOVA	Wilcoxon rank sum test		
		SIRS	Pneumonia	Sepsis
ISS	70.26	0.017	0.435	2.05·10^-5^
AIS Head	7.16	0.837	2.99·10^-4^	0.220
AIS Thorax	3.47	0.214	0.141	0.025
AIS Abdomen	5.53	0.478	0.720	0.560
AIS Extremities	3.66	0.148	0.207	0.002
GCS	32.87	4.71·10^-4^	1.54·10^-4^	1.81·10^-4^
APACHE II score	19.60	0.267	0.001	4.11·10^-4^
SOFA score	11.27	0.195	0.023	0.022
IL-6	19.90	0.602	0.032	3.26·10^-4^
IL-10	11.52	0.662	0.164	0.002
AP	2.01	0.571	0.034	0.109
GLDH	5.25	0.977	0.581	0.030
GGT	3.13	0.647	0.013	0.705
GOT	17.07	0.079	0.924	5.04·10^-4^
GPT	12.52	0.412	0.857	0.003
Leukocytes	5.73	0.031	0.589	0.947
CRP	2.59	0.346	0.762	0.248
Lactate	4.72	0.991	0.211	0.061
PT	27.05	0.010	0.305	4.81·10^-6^
INR	27.33	0.011	0.267	5.61·10^-6^
PTT	31.25	0.016	0.001	2.24·10^-6^
Fibrinogen	1.75	0.476	0.784	0.265
Platelets	7.40	0.261	0.071	0.028
Na+	5.16	0.110	0.254	0.138
K+	5.92	0.267	0.868	0.015
Creatinin	7.19	0.541	0.421	0.035
Hematocrit	19.70	0.026	0.121	6.65·10^-5^
Bilirubin	2.20	0.377	0.319	0.383
Hb	28.07	0.011	0.024	1.98·10^-6^
Features that reject the null hypothesis	13/29	8/29	9/29	19/29

The green table color indicates a rejection of the null hypothesis, whereas the red table coloring indicates that there is not enough evidence to reject the null hypothesis. The χ2-values from the Kruskal-Wallis ANOVA test and p-values from the Wilcoxon rank sum test for the different subgroups are given as table entries. AIS, abbreviated injury scale; AP, alkaline phosphatase; APACHE, acute physiology and chronic health evaluation; CRP, C-reactive protein; GCS, glasgow coma scale; GLDH, glutamate dehydrogenase; GGT, gamma glutamyl transpeptidase; GOT, glutamic oxaloacetic transaminase; GPT, glutamic pyruvic transaminase; Hb, hemoglobin; IL, interleukin; INR, international normalized ratio; ISS, injury severity score; K, potassium; Na, sodium; PT, prothrombin time; PTT, partial thromboplastin time; SOFA, sequential organ failure assessment.

### 3.3 Cluster analysis revealed distinct biomarker patterns for hyperinflammatory and infectious complications

To identify the number of patient risk clusters, present during the 3 days after injury, 317 patients admitted to the ICU after trauma were subjected to filtered k-means clustering. This analysis yielded four major cluster groups that were distinguished by distinct inflammatory profiles for days 0-3 post-injury. These clustering techniques produced the following patient groups: a favorable outcome group with “no complications” (cluster 1), a SIRS group with high inflammatory load (cluster 3) and unfavorable infectious outcome groups suffering from sepsis or pneumonia (cluster 2 and 4) (**Table 3** and **Supplementary Table S1**).

**Table 3:** Overview of polytrauma patient cohort and associated injury patterns.

Attribute	Full data (n=317)	Cluster 1 (n=101)	Cluster 2 (n=99)	Cluster 3 (n=58)	Cluster 4 (n=59)	p<0.05
ISS (–)	26.35	20.97	28.05	36	23.27	b
AIS Head (–)	2.27	2.23	0.31	3.67	4.25	e
AIS Thorax (–)	2.01	1.34	3.18	3.17	0.09	n.s.
AIS Abdomen (–)	0.76	0.51	1.52	0.71	0	e
AIS Extremities (–)	1.15	0.96	1.53	1.46	0.52	n.s.
GCS (–)	8.45	14.32	7.68	3.56	4.54	b
APACHE II score (–)	13.12	7.06	15.91	16.38	15.58	n.s.
SOFA score (–)	4.11	1.66	5.56	5.73	4.24	b
IL-6 (pg/ml)	165.81	60.98	257.93	229.61	127.98	a
IL-10 (pg/ml)	43.26	24.78	57.86	56.52	37.37	n.s.
AP (U/l)	47.97	52.98	43.16	44.70	50.68	n.s.
GLDH (U/l)	18.01	8.32	29.83	22.95	9.91	a
GGT (U/l)	38.68	45.46	36.23	27.20	42.47	b
GOT (U/l)	97.11	53.25	169.71	97.08	50.40	f
GPT (U/l)	63.98	40.33	101.23	75.03	31.09	f
Leukocytes (U/nl)	10.24	10.00	9.74	10.47	11.24	f
CRP (mg/dl)	3.21	3.36	2.52	3.52	3.78	f
Lactate (mg/dl)	19.29	16.73	21.68	19.85	19.13	a
PT (%)	84.53	91.42	79.55	79.57	85.96	n.s.
INR (–)	1.16	1.09	1.22	1.21	1.16	a
PTT (s)	31.31	27.49	33.76	34.01	31.13	b
Fibrinogen (mg/dl)	309.87	307.52	330.20	308.32	281.32	f
Platelets (cells/nl)	168.19	185.12	151.84	157.57	176.61	a
Na+ (mmol/l)	143.19	139.55	145.03	142.65	146.87	c
K+ (mmol/l)	4.07	4.01	4.18	4.04	4.06	a
Creatinine (mg/dl)	0.92	0.83	1.02	0.91	0.92	a
Hematocrit (%)	31.02	34.56	28.36	29.25	31.17	a
Bilirubin (mg/dl)	0.66	0.66	0.69	0.62	0.67	d
Hb (g/dl)	10.41	11.69	9.49	9.85	10.29	a

The cluster numbers allocate to the following complication patterns: 1 – no complications, 2 – sepsis, 3 – systemic inflammatory response syndrome (SIRS), and 4 – pneumonia. AIS, abbreviated injury scale; AP, alkaline phosphatase; APACHE, acute physiology and chronic health evaluation; CRP, C-reactive protein; dl, deciliter; g, gram; GCS, glasgow coma scale; GLDH, glutamate dehydrogenase; GGT, gamma glutamyl transpeptidase; GOT, glutamic oxaloacetic transaminase; GPT, glutamic pyruvic transaminase; Hb, hemoglobin; IL, interleukin; INR, international normalized ratio; ISS, injury severity score; K, potassium; l, liter; mg, milligram; ml, milliliter; mmol, millimoles; Na, sodium; nl, nanoliter; n.s., not significant; pg, picogram; PT, prothrombin time; PTT, partial thromboplastin time; SOFA, sequential organ failure assessment; U, units. Significant differences (p <0.05) between the groups are indicated as follows: a: cluster 1 vs. cluster 2, b: cluster 1 vs. cluster 3, c: cluster 1 vs. cluster 4, d: cluster 2 vs. cluster 3, e: cluster 2 vs. cluster 4, f: cluster 3 vs. cluster 4.

**Figure 3:**
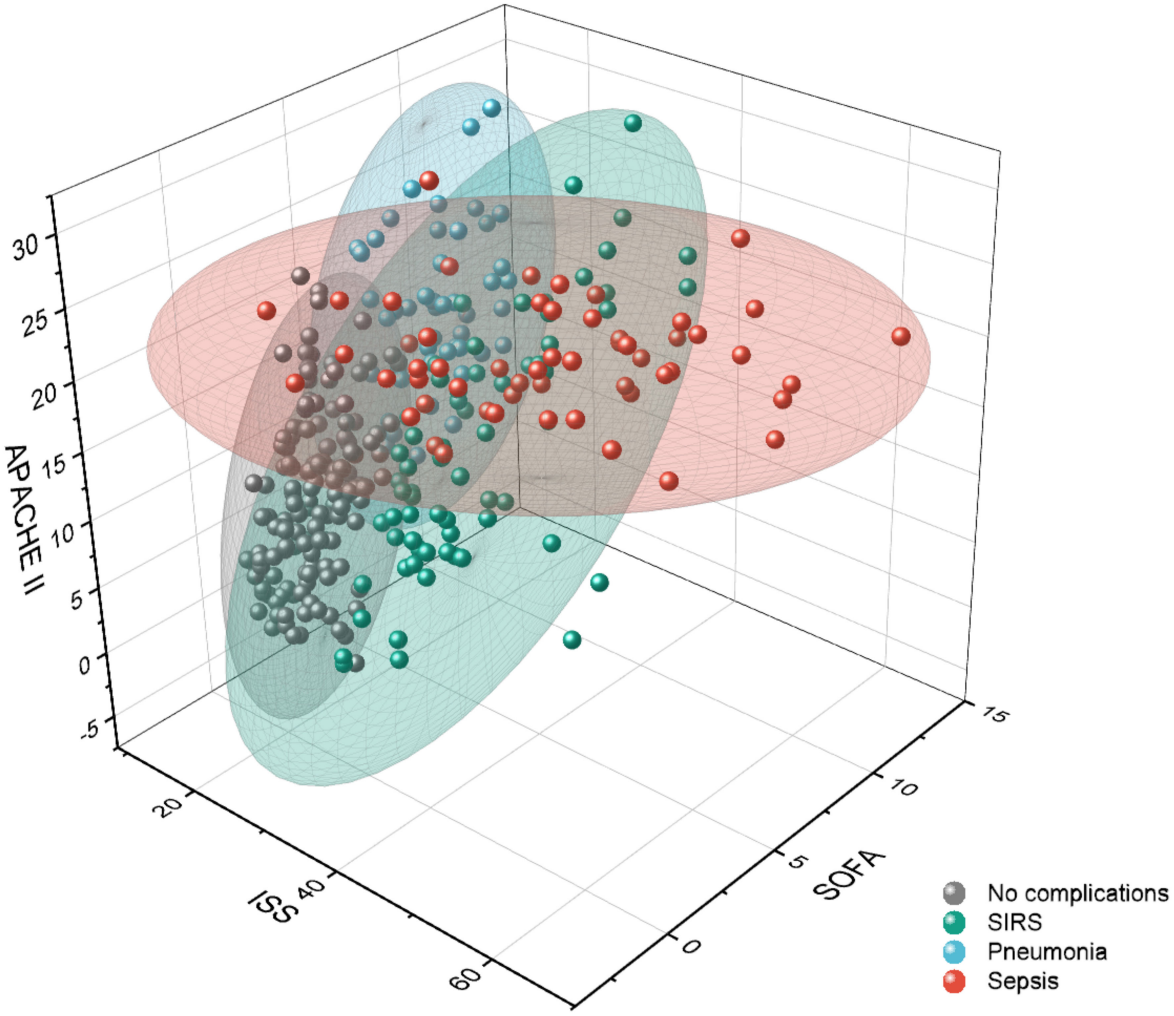
Visualization of outcome-related patient clusters with respect to clinical scores: the total injury severity represented by the injury severity score (ISS) and the disease severity scores acute physiology and chronic health evaluation (APACHE) II and sequential organ failure assessment (SOFA). Data points represent the individual patients, and the colored ellipsoids represent the cluster areas. SIRS, systemic inflammatory response syndrome.

In the following, we discuss the unique parameter differences and additional clinical considerations for each respective patient cluster and their associated outcome risk. The identified cluster revealed a very distinct injury pattern among each other (**Figure 3**).

Patients that belonged to cluster 1 (n=101) were mainly associated to the sub-group showing no complications. Therefore, the AIS related to different body parts, the disease severity scores APACHE II and SOFA, and the cytokines IL-6 and IL-10 are significantly lower than the average of the overall patient cohort (**Table 3**). The 99 patients clustered in cluster 2, where the majority of septic patients was observed, showed a low AIS of the head (0.31 points) and a high AIS of the thorax (3.18 points) with significantly elevated level of IL-6 (257.93 pg/ml), GPT (101.23 U/l) and GOT (169.71 U/l) compared to the centroid of the full data set (**Table 3**). In comparison to the patients in cluster 2 with high thorax and low head injury, 58 patients in cluster 3 had high AIS for head (3.67 points) and thorax (3.17 points) injury and showed mainly a pro-inflammatory complication pattern (SIRS) accompanied with higher cytokine levels (IL-6 concentration of 229.61 pg/ml and IL-10 concentration of 56.52 pg/ml) and disease severity scores (APACHE II of 16.38 points and SOFA of 5.73) above average (**Table 3**). 59 patients in cluster 4 have significantly higher head injury scores (AIS Head with 4.25 points) whereas the other body regions were mostly without traumatic injuries and showed pneumonia as a main complication class (**Table 3**). The patients with an isolated traumatic brain injury in cluster 4 had lower cytokine levels (IL-6 concentration of 127.98 pg/ml and IL-10 concentration of 37.37 pg/ml) and lower levels of biomarkers related to the liver function (GLDH, GGT, GOT, GPT) that were comparable with cluster 1 (mainly patients with no complications) (**Table 3**).

### 3.4 Ensemble classifiers predicted adverse outcomes with high accuracy

Classification analysis was conducted on the patient cohort illustrated in **Figure 1** with the demographics and outcomes given in **Table 1**. The patient subgroup with SIRS was neglected in the classification due to their relatively low impact on adverse outcome patterns (**Figure 2**) and the weakest correlation observed in the Wilcoxon rank sum test compared to the sub-group that showed no complications (**Table 2**). The patient sub-group with pneumonia and sepsis are combined in an “infectious complication” group since more than 72% of the patients with sepsis also developed pneumonia during their hospitalization. For the classification of polytrauma patients in risk groups (no complications, infectious complication) several classifiers (naive bayes, random forest and ensemble classifiers) were optimized to achieve a high F1 score and best receiver operating characteristics (ROC) (**Supplementary Tables S2**, **S3**).

**Figure 4:**
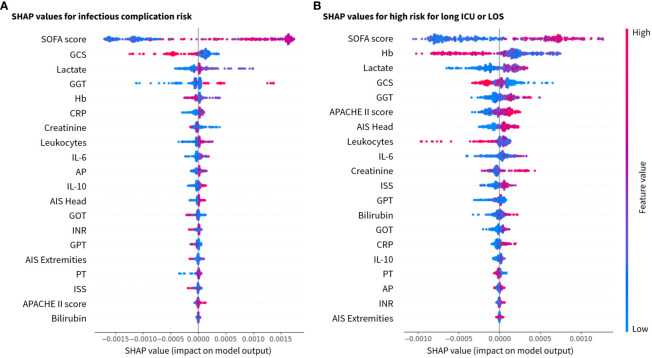
SHAP values for the 20 most relevant features in the XGBoost model for the investigated class labels **(A)** for infection risk, and **(B)** risk for long stay (ICU and total hospital stay) ranked by mean absolute values. x-axis: SHAP values and y-axis: features ranked by importance. AIS (–), abbreviated injury scale; AP (U/l), alkaline phosphatase; APACHE, acute physiology and chronic health evaluation; CRP (mg/dl), C-reactive protein; GCS, glasgow coma scale; GGT (U/l), gamma glutamyl transpeptidase; GOT (U/l), glutamic oxaloacetic transaminase; GPT (U/l), glutamic pyruvic transaminase; Hb (g/dl), hemoglobin; IL(pg/ml), interleukin; INR, international normalized ratio; ISS, injury severity score; PT (%), prothrombin time; SOFA, sequential organ failure assessment.

**Figure 5:**
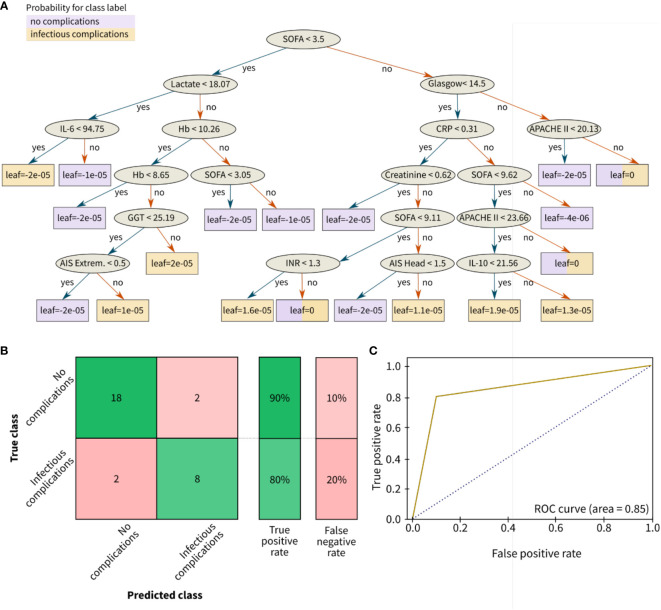
Results for the classification of infectious outcomes in polytrauma patients. **(A)** Decision tree, **(B)** Confusion matrix, and **(C)** Receiver operating characteristic (ROC) curve for risk classification with the following outcome groups as classes: group 1 (with no complications) and group 2 with infectious complications (pneumonia and/or sepsis). The leaves of **(A)** correspond to the final nodes of the decision tree where the data does not split any further and points to the predicted risk classes of the respective patient cohort: no complications (purple) and infectious complications (yellow). The prediction accuracy between the true class (columns) and the predicted class (rows) is given in % as true positive or false negative rate. The confusion matrix for the classification problem was achieved by the ensemble learning algorithm XGBoost. AIS, abbreviated injury scale; APACHE, acute physiology and chronic health evaluation; CRP (mg/dl), C-reactive protein; Extrem., extremities; Glasgow, glasgow coma scale; GGT (U/l), gamma glutamyl transpeptidase; Hb (g/dl), hemoglobin; IL (pg/ml), interleukin; INR, international normalized ratio; SOFA, sequential organ failure assessment.

The best classification performance was achieved with the ensemble classifier XGBoost, a boosting algorithm designed to turn week classifiers such as decision trees as base classifiers into an ensemble of strong classifiers. XGBoost performs well even for imbalanced class problems. The biomarker predictor ranking was analyzed by gradient boosting and features governing these results are explained using SHAP values (**Figure 4**). Based on boosting ensemble classification XGBoost, 18 from 20 patients (90%) in the test set were correctly classified as low-risk group (no complications). For the high-risk groups associated with development of pneumonia and sepsis, 8 from 10 patients in the test set (80%) were correctly identified (**Figure 5**).

The classification results were explained by SHAP values, which calculate the importance of a feature by comparing the model predictions with and without the selection of certain features. Based on the explainability analysis with SHAP values, the clinical and immunological trauma features that had the largest importance on the model performance were identified. The top clinical feature for both investigated risk scenarios (infectious complications and long ICU stay) were SOFA score, GCS, Lactate, GGT and Hb in a slightly varying order (**Figure 4**). Furthermore, the immunological features of total leukocyte counts and IL-6 concentration were found to be top ranking in both scenarios, whereas the anti-inflammatory cytokine IL-10 had a higher feature importance in the infectious complication patterns than in the length of stay risk prediction.

**Figure 5** is showing the resulting decision tree after ten learning cycles (M = 10) with a learning rate of 0.001. According to the feature ranking, the disease severity scores SOFA score and GCS, the injury-related scores such as ISS and AIS, the anti-inflammatory biomarker IL-10, the liver function parameters (GGT and GOT) as well as bleeding and coagulation-related parameters (Hb) were branches of the decision tree (**Figure 5**).

**Figure 6:**
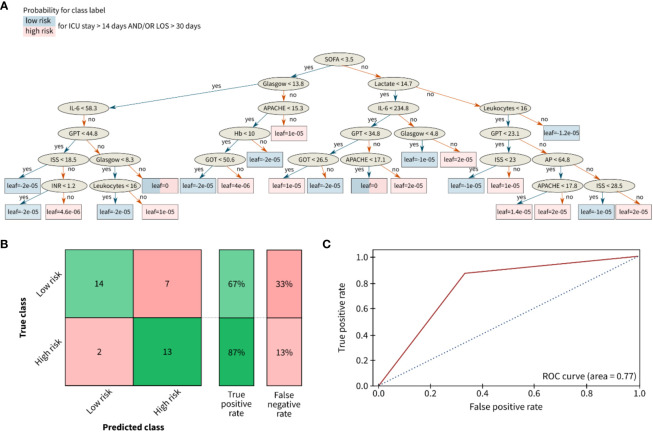
Results for the classification of the length of stay of polytrauma patients in the ICU or the total hospital stay. **(A)** Decision tree, **(B)** Confusion matrix, and **(C)** Receiver opertainig characteristic (ROC) curve for risk classification with the following outcome groups as classes: group 1 (with low risk) and group 2 with high risk for a long LOS. The leaves of **(A)** correspond to the final nodes of the decision tree where the data does not split any further and points to the predicted risk classes of the respective patient cohort: low risk (blue) and high risk (red). The prediction accuracy between the true class (columns) and the predicted class (rows) is given in % as true positive or false negative rate. The confusion matrix for the classification problem was achieved by the ensemble learning algorithm XGBoost. AP (U/l), alkaline phosphatase; APACHE, acute physiology and chronic health evaluation; Glasgow, glasgow coma scale; GOT (U/l), glutamic oxaloacetic transaminase; GPT (U/l), glutamic pyruvic transaminase; Hb (g/dl), hemoglobin; IL (pg/ml), interleukin; INR, international normalized ratio; ISS, injury severity score; SOFA, sequential organ failure assessment.

In comparison to the infectious classification scenario, the prediction accuracy for the length of stay risk classification (ICU stay for >14 days and/or LOS for >30 days) slightly decreased to 67% for the low-risk group and 87% for the high-risk group (**Figure 6**).

## 4 Discussion

The objective of this study was to use various machine learning algorithms and explainability based on SHAP values based on clinical features collected in the early hospitalization phase to identify clinical and inflammatory biomarker in a polytrauma study cohort with various injury patterns. The focus was on adverse outcome patterns such as SIRS and infectious complications like pneumonia and sepsis, analyzing feature patterns within three days post-injury. Through filtered k-means clustering, four distinct clusters with different injury and feature patterns were identified. These clusters exhibited variations in clinical features and inflammatory profiles upon admission and during the initial three days post-injury.

Regarding the risk classification of patient sub-groups, the results showed that ensemble-based methods outperformed naive Bayes or random forest classifiers in terms of accuracy. The best classification performance was achieved with XGBoost, a boosting algorithm designed to turn week classifiers such as decision trees as base classifiers into an ensemble of strong classifiers. XGBoost is an ensemble approach which is efficient in predicting the plausible classes accurately since sometimes only base classifier alone cannot classify the class labels and the predictions can be biased. Gradient boosting helps in overcoming this effect by building sequence of base classifiers such that each successor aims in reducing the error of the predecessor employing a gradient descent approach. Extreme gradient boosting is a type of gradient boosting approach which uses second order optimization function to optimize the errors in the predictions (24). The exact form of the pseudo-loss is under control of the algorithm so that the weak classifier can focus also on the groups which are hardest to distinguish from the correct group. The XGBoost ensemble classifier achieved 90% accuracy in classifying patients without complications and 80% accuracy in identifying patients with infectious complications (pneumonia and sepsis). The higher rate of misclassified cases in the pneumonia and sepsis group may be attributed to complex comorbidities and medication history of these patients. Due to the relatively small sample size of patients with sepsis (n=33) and pneumonia (n=39), future validation studies in larger cohorts and a rigorous study designs are needed to enhance classification accuracy further.

Even though disease severity scores are not the key elements of treatment, they are however, an essential part of improvement in clinical decisions and in identifying patients with unexpected outcomes, such as the investigated subjective score variables APACHE II and SOFA that are collected daily in our study (25).Consistent with the previous results of Tranca et al. (2016), the ensemble classifier predicts that most patients with a SOFA cut-off score below 3.45 points did not develop sepsis (26). Moreover, in line with Tranca et al. (2016) (26), it was confirmed that patients with APACHE II score below 10 points did not develop sepsis and indicate a discrimination between the pneumonia subgroup from patients without complications. Based on the AIS head, a cut-off score of AIS head=1.5 in combination with other predictive features were found to separate low-risk from the high-risk patient sub-group.

Consistent with previous studies, this prospective machine learning study also highlighted the importance of measuring pro- and anti-inflammatory cytokines (e.g. IL-6 and IL-10) for risk classification in trauma patients (27, 28). The immunological features of total leukocyte counts and IL-6 concentration were found to be top ranking in both scenarios (infectious complications and long ICU stay), whereas the anti-inflammatory cytokine IL-10 had a higher feature importance in the infectious complication patterns than in the length of stay risk prediction. Together, elevated IL-6 levels (>95 pg/ml) and decreased IL-10 levels (<21 pg/ml) in combination with other features were predictive of infectious risk classification. This study demonstrated the potential for early risk stratification of severely injured trauma patients into sub-groups at risk for specific clinical trajectories. This approach may aid in tailored research and clinical therapies for polytrauma patients, aligning with the findings of Liu et al. (2020) (7). By utilizing the explainability analysis based on SHAP values, it was elucidated how clinical and immunological biomarkers that were routinely collected during ICU admission impact the decision-making process of black-box machine learning models. Thus, the present study validated the clinical predictors for infectious complications and the LOS in the hospital.

Some additional features including the importance of GGT as a feature for clustering remain intriguing. Fisher et al. have shown that GGT as a useful and simple biomarker at admission is among the independent indicators and predictors of in-hospital mortality in older hip fracture patients (29). In the realm of clinical models, the role of GGT is intricate, and subject to various factors and contextual nuances. GGT, an enzyme distributed in the liver, biliary tract, and diverse tissues, can undergo elevation in response to a spectrum of medical conditions, encompassing liver damage, alcohol consumption, and cholestatic liver disease (30, 31). Its levels can also be influenced by medications and concurrent health issues. To discern why GGT assumes a noteworthy role in this study, several key considerations come into play. It is essential to note that individuals with a documented history of chronic diseases were deliberately excluded from this study. However, the impact of GGT in this model may hinge on the timing of measurements. The model employs the mean GGT value from the first three days post-admission. Nonetheless, the fluctuating nature of GGT levels over time should be acknowledged, as elevated levels can signify distinct conditions at different stages of illness. Moreover, undisclosed pre-existing medical conditions and concurrent health issues among patients could significantly impact GGT levels and their clinical relevance, potentially confounding the results. Understanding the interrelationships between GGT, GOT, GPT, bilirubin levels, and other markers of liver function is pivotal. These relationships could offer a more comprehensive understanding of liver health and potential causes of their elevation, since notably, these markers rank among the top 20 most relevant features for classification in our model.

Our study contains several limitations. The sepsis sub-group meets the SIRS criteria, with the added specificity of including individuals with bloodstream infections. It’s important to note that the sepsis sub-group also encompasses patients with pneumonia who developed sepsis, whereas the pneumonia group does not involve sepsis, and the SIRS group excludes both pneumonia and sepsis cases. It must be acknowledged that a re-classification of patients according to the more recent sepsis-3 definition will be of higher clinical relevance (32). A retrospective re-classification of patients from the present study according to sepsis-3 criteria was performed, however, there are serious limitations in the patient records, which only provided a single daily recording of the worst SOFA score for ten consecutive days post-hospital admission. Thus, the retrospective re-classification according to sepsis-3 criteria referred only to SOFA score changes of ≥2 points within 24 hours. Despite this challenge, all 33 septic patients in the presented sub-group do meet the sepsis-3 criteria based on the records. This data should not be overinterpreted, although Kim et al. have reported that some validity on the assessment of the prognostic accuracy of the initial SOFA score at the time of sepsis recognition which was lower than the 24-h maximal SOFA score in ED patients with septic shock (33). Their insights, while valuable, emphasize the need for continuous monitoring of SOFA scores over an extended period. Our study, on the other hand, contends that assessing the worst SOFA score within 24 hours may capture a critical moment in a patient’s condition, offering practicality in historical data analyses. While the retrospective nature of such approaching definitely poses limitations, the assessment of the worst SOFA score within 24 hours during the hospital stay may provide some clinically relevant information. Yet, clearly, prospective studies with continuous SOFA scoring during the progression of sepsis are crucial for a more comprehensive understanding, and we acknowledge the need for further validation through such studies.

In addition, the distinction between the sepsis group, characterized by systemic infection, and the pneumonia group, where bacteriological diagnosis primarily resides in the lungs, underscores the complexity of infections in trauma patients. A pivotal question emerges regarding the utility of antibiotic treatment initiation on a specific day as a surrogate for infection. The lack of valid and reliable records on antibiotic regimens in the assessed patient cohort complicates such assessment. Sepsis, a complex condition, may not always necessitate bacteriological confirmation, as demonstrated in the present study where a bacteriological diagnosis was consistently found. Its definition has evolved to reflect a dysregulated host response to infection, irrespective of the infectious agent’s nature—bacteria, viruses, fungi, or even non-infectious triggers. While bacteriological confirmation holds value, sepsis diagnosis relies significantly on clinical presentation and established criteria encompassing signs such as body temperature fluctuations, increased heart rate, and abnormal white blood cell counts (32). The notion of using the initiation of antibiotic treatment on a certain day as a surrogate for infection poses challenges. Prophylactic antibiotic use is crucial in certain trauma scenarios, such as traumatic brain injuries (TBI), penetrating injuries, open fractures, and high-risk orthopedic procedures (34). However, initiating antibiotics early, though important in suspected infections, does not inherently confirm the presence of an infection, and must be critically discussed in terms of their role in the development of post-traumatic infections and microbial selection (34). Empirical antibiotic initiation in critically ill patients is often a necessity, with subsequent treatment decisions guided by culture results and clinical response. Clinical judgment, complemented by other diagnostic modalities such as blood cultures, imaging studies, and biomarkers, should collectively guide the diagnosis and management of sepsis and associated infections in trauma patients. In addition, trauma patients, particularly those with brain injuries, may face an elevated risk of developing ventilator-associated pneumonia (VAP) (35), confirming the data from the present study revealing higher AIS head values in patients with pneumonia compared to other groups. Thus, multiple factors including compromised immune function, prolonged mechanical ventilation, impaired airway protection mechanisms but also the injury patterns demand a careful management including antibiotics regimes to prevent complications such as VAP in trauma patients.

The model development process and findings in the present study could be employed to predict the clinical course and identify high-risk individuals for inflammatory or infectious complications among severely injured trauma patients. The presented ensemble methods identified key features in polytraumatized patients, that allowed to predict patient`s outcomes. Thus, implementing such models may enhance clinical decision-making by enabling personalized treatment strategies based on individual risk profiles in future.

## Data availability statement

The raw data supporting the conclusions of this article will be made available by the authors, without undue reservation.

## Ethics statement

The studies involving humans were approved by University Hospital Frankfurt of the Goethe University (nr.: 312/10). The studies were conducted in accordance with the local legislation and institutional requirements. Written informed consent for participation in this study was provided by the participants’ legal guardians/next of kin.

## Author contributions

MF: Conceptualization, Data curation, Formal Analysis, Investigation, Methodology, Software, Writing – original draft. RM: Formal Analysis, Investigation, Methodology, Visualization, Writing – review & editing. CB: Formal Analysis, Validation, Writing – review & editing. IM: Funding acquisition, Resources, Writing – review & editing. CH: Resources, Writing – review & editing. BR: Conceptualization, Data curation, Formal Analysis, Funding acquisition, Investigation, Methodology, Project administration, Resources, Supervision, Validation, Writing – original draft.

## Supplementary material

Supplementary Table 1 Misclustered instances from filtered clustering based on k-means algorithm. Abbreviation: SIRS, systemic inflammatory response syndrome.Supplementary information for classification analysis

Supplementary Table 2 Accuracy of different classifiers for infectious complications.

Supplementary Table 3 Accuracy of different classifiers for risk of long intensive care unit (ICU) and length of stay (LOS) prediction.

Supplementary Figure 1 Box plots of outcome associated parameters related to the complication risk. Lower and upper box boundaries correspond to 25th and 75th percentiles, respectively. The line inside represents the box median. The lower and upper error lines (whiskers) correspond to the 10th and 90th percentiles, respectively. The asterisk next to the 10th and 90th percentiles indicate statistical significance between the complication group and the patient group having no complications. Points above and below the whiskers indicate outliers outside the 10th and 90th percentiles. Abbreviations: APACHE, acute physiology and chronic health evaluation; SIRS, systemic inflammatory response syndrome; SOFA, sequential organ failure assessment.

Supplementary Figure 2 Box plots of cytokines and predictive markers related to the complication risk. Lower and upper box boundaries correspond to 25th and 75th percentiles, respectively. The line inside represents the box median. The lower and upper error lines (whiskers) correspond to the 10th and 90th percentiles, respectively. The asterisk next to the 10th and 90th percentiles indicate statistical significance between the complication group and the patient group having no complications. Points above and below the whiskers indicate outliers outside the 10th and 90th percentiles. Abbreviations: IL, interleukin; PCT, procalcitonin; SIRS, systemic inflammatory response syndrome.

Supplementary Figure 3 Box plots of serum markers for liver function related to the complication risk. Lower and upper box boundaries correspond to 25th and 75th percentiles, respectively. The line inside represents the box median. The lower and upper error lines (whiskers) correspond to the 10th and 90th percentiles, respectively. The asterisk next to the 10th 29 and 90th percentiles indicate statistical significance between the complication group and the patient group having no complications. Points above and below the whiskers indicate outliers outside the 10th and 90th percentiles. Abbreviations: AP, alkaline phosphatase; CRP, C-reactive protein; GLDH, glutamate dehydrogenase; GGT, gamma glutamyl transpeptidase; GOT, glutamic oxaloacetic transaminase; GPT, glutamic pyruvic transaminase.

Supplementary Figure 4 Box plots of serum markers for coagulation factors related to the complication risk. Lower and upper box boundaries correspond to 25th and 75th percentiles, respectively. The line inside represents the box median. The lower and upper error lines (whiskers) correspond to the 10th and 90th percentiles, respectively. The asterisk next to the 10th and 90th percentiles indicate statistical significance between the complication group and the patient group having no complications. Points above and below the whiskers indicate outliers outside the 10th and 90th percentiles. Abbreviations: INR, international normalized ratio; PTT, partial thromboplastin time. Supplementary information for statistical tests and correlation analysis

Supplementary Figure 5 Feature importance ranking based on the F1 score with respect to the two investigated risk scenarios: (A) Risk for infectious complications, and (B) risk for a long stay in the intensive care unit (ICU) or total length of stay (LOS) in hospital. Abbreviations: Abbreviations: AIS, abbreviated injury scale; APACHE, acute physiology and chronic health evaluation; GGT, gamma glutamyl transpeptidase; GOT, glutamic oxaloacetic transaminase; GPT, glutamic pyruvic transaminase; SOFA, sequential organ failure assessment. GGT - Gamma glutamyl transpeptidase, GOT-Glutamic oxaloacetic transaminase, GPT-Glutamic pyruvic transaminase. Supplementary information for clustering analysis
