# Supplementary material for: Utilizing predictive machine-learning modelling unveils feature-based risk assessment system for hyperinflammatory patterns and infectious outcomes in polytrauma
Source: Front Immunol. 2023 Dec 12;14:1281674. doi: 10.3389/fimmu.2023.1281674 (PMC10773821; doi:10.3389/fimmu.2023.1281674)
Supplement: Supplementary Table 1 — Misclustered instances from filtered clustering based on k-means algorithm. Abbreviation: SIRS, systemic inflammatory response syndrome.Supplementary information for classification analysis [file Table_1.docx]

| **Attribute** | **Cluster** | | | |
| --- | --- | --- | --- | --- |
|  | **1** | **2** | **3** | **4** |
| No complications | 85 | 53 | 26 | 30 |
| SIRS | 10 | 18 | 4 | 9 |
| Pneumonia | 4 | 8 | 9 | 18 |
| Sepsis | 2 | 20 | 9 | 2 |
