# Supplementary material for: Utilizing predictive machine-learning modelling unveils feature-based risk assessment system for hyperinflammatory patterns and infectious outcomes in polytrauma
Source: Front Immunol. 2023 Dec 12;14:1281674. doi: 10.3389/fimmu.2023.1281674 (PMC10773821; doi:10.3389/fimmu.2023.1281674)
Supplement: Supplementary Table 2 — Accuracy of different classifiers for infectious complications. [file Table_2.docx]

| **Model** | **Score** | | |  |
| --- | --- | --- | --- | --- |
|  | **Accuracy** | **F1** | **AUC** | |
| XGBoost | 0.87 | 0.80 | 0.85 | |
| Random forest | 0.73 | 0.55 | 0.67 | |
| Naive bayes | 0.66 | 0.37 | 0.57 | |
