# Supplementary material for: Utilizing predictive machine-learning modelling unveils feature-based risk assessment system for hyperinflammatory patterns and infectious outcomes in polytrauma
Source: Front Immunol. 2023 Dec 12;14:1281674. doi: 10.3389/fimmu.2023.1281674 (PMC10773821; doi:10.3389/fimmu.2023.1281674)
Supplement: Supplementary Table 3 — Accuracy of different classifiers for risk of long intensive care unit (ICU) and length of stay (LOS) prediction. [file Table_3.docx]

| **Model** | **Score** | | |  |
| --- | --- | --- | --- | --- |
|  | **Accuracy** | **F1** | **AUC** | |
| XGBoost | 0.75 | 0.74 | 0.76 | |
| Random forest | 0.55 | 0.5 | 0.55 | |
| Naive bayes | 0.55 | 0.42 | 0.53 | |
