# Supplementary figures and images for: Utilizing predictive machine-learning modelling unveils feature-based risk assessment system for hyperinflammatory patterns and infectious outcomes in polytrauma
Source: Front Immunol. 2023 Dec 12;14:1281674. doi: 10.3389/fimmu.2023.1281674 (PMC10773821; doi:10.3389/fimmu.2023.1281674)

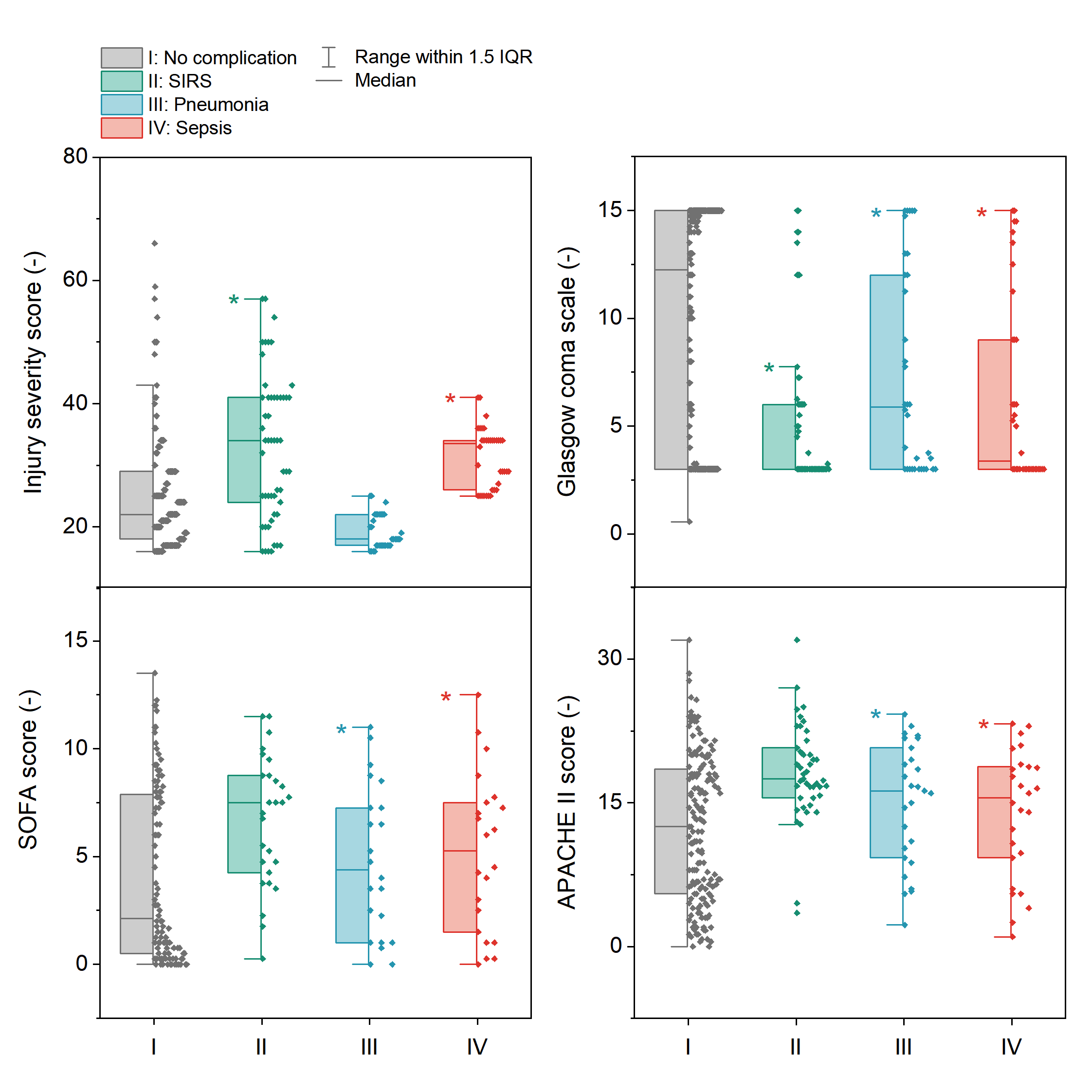

Supplement: Supplementary Figure 1 — Box plots of outcome associated parameters related to the complication risk. Lower and upper box boundaries correspond to 25th and 75th percentiles, respectively. The line inside represents the box median. The lower and upper error lines (whiskers) correspond to the 10th and 90th percentiles, respectively. The asterisk next to the 10th and 90th percentiles indicate statistical significance between the complication group and the patient group having no complications. Points above and below the whiskers indicate outliers outside the 10th and 90th percentiles. Abbreviations: APACHE, acute physiology and chronic health evaluation; SIRS, systemic inflammatory response syndrome; SOFA, sequential organ failure assessment. [file Image_1.tiff]

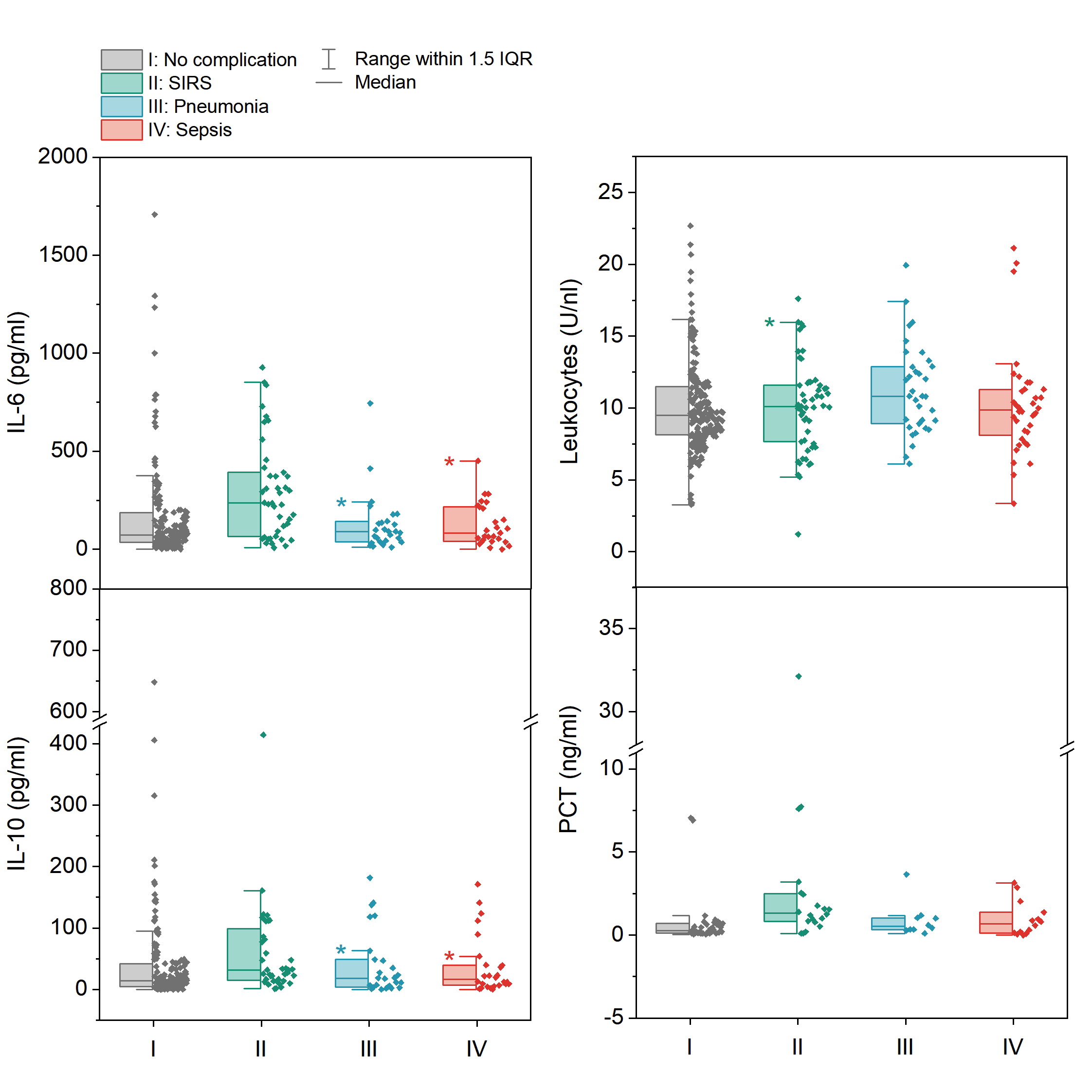

Supplement: Supplementary Figure 2 — Box plots of cytokines and predictive markers related to the complication risk. Lower and upper box boundaries correspond to 25th and 75th percentiles, respectively. The line inside represents the box median. The lower and upper error lines (whiskers) correspond to the 10th and 90th percentiles, respectively. The asterisk next to the 10th and 90th percentiles indicate statistical significance between the complication group and the patient group having no complications. Points above and below the whiskers indicate outliers outside the 10th and 90th percentiles. Abbreviations: IL, interleukin; PCT, procalcitonin; SIRS, systemic inflammatory response syndrome. [file Image_2.tiff]

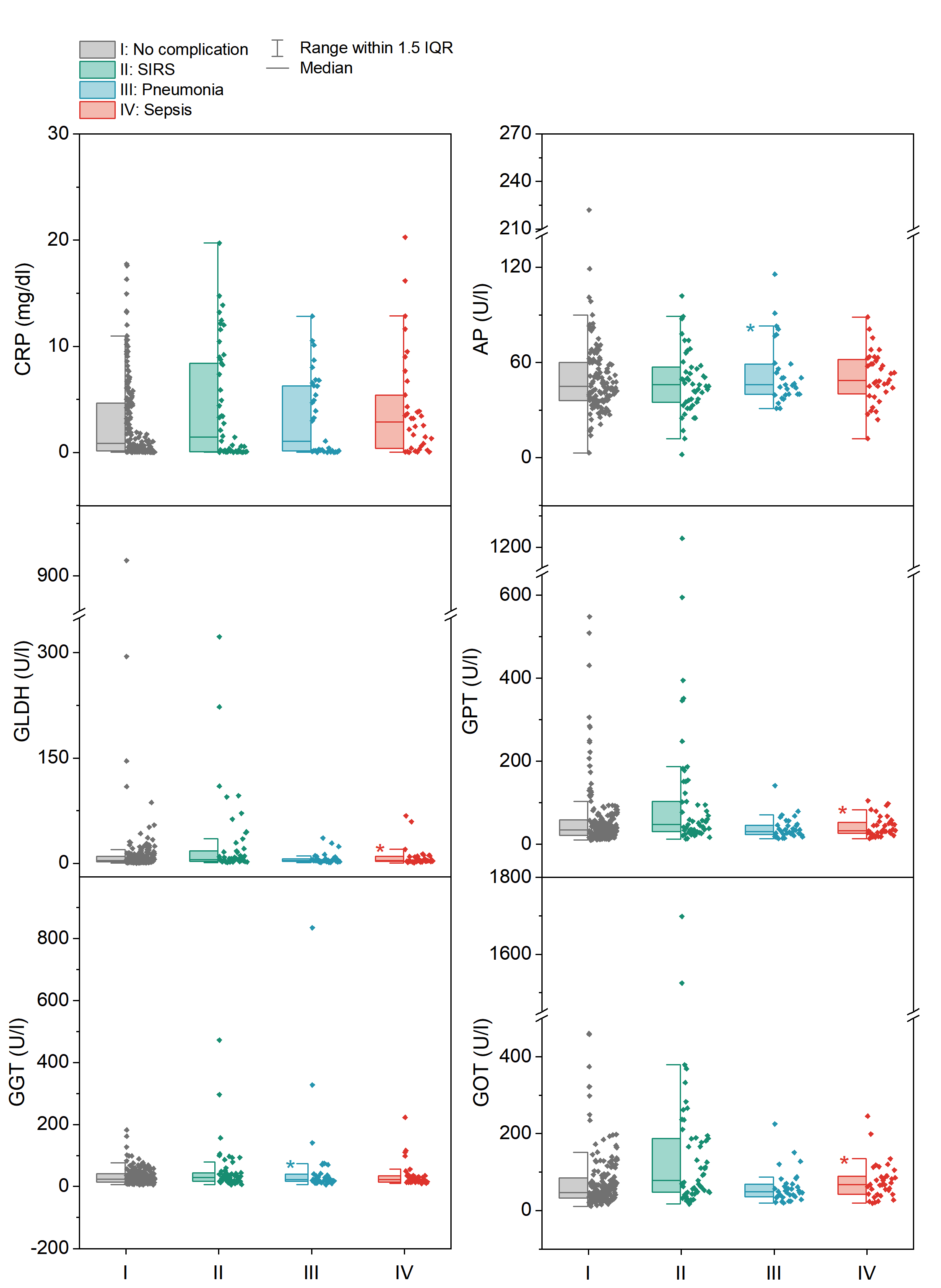

Supplement: Supplementary Figure 3 — Box plots of serum markers for liver function related to the complication risk. Lower and upper box boundaries correspond to 25th and 75th percentiles, respectively. The line inside represents the box median. The lower and upper error lines (whiskers) correspond to the 10th and 90th percentiles, respectively. The asterisk next to the 10th 29 and 90th percentiles indicate statistical significance between the complication group and the patient group having no complications. Points above and below the whiskers indicate outliers outside the 10th and 90th percentiles. Abbreviations: AP, alkaline phosphatase; CRP, C-reactive protein; GLDH, glutamate dehydrogenase; GGT, gamma glutamyl transpeptidase; GOT, glutamic oxaloacetic transaminase; GPT, glutamic pyruvic transaminase. [file Image_3.tiff]

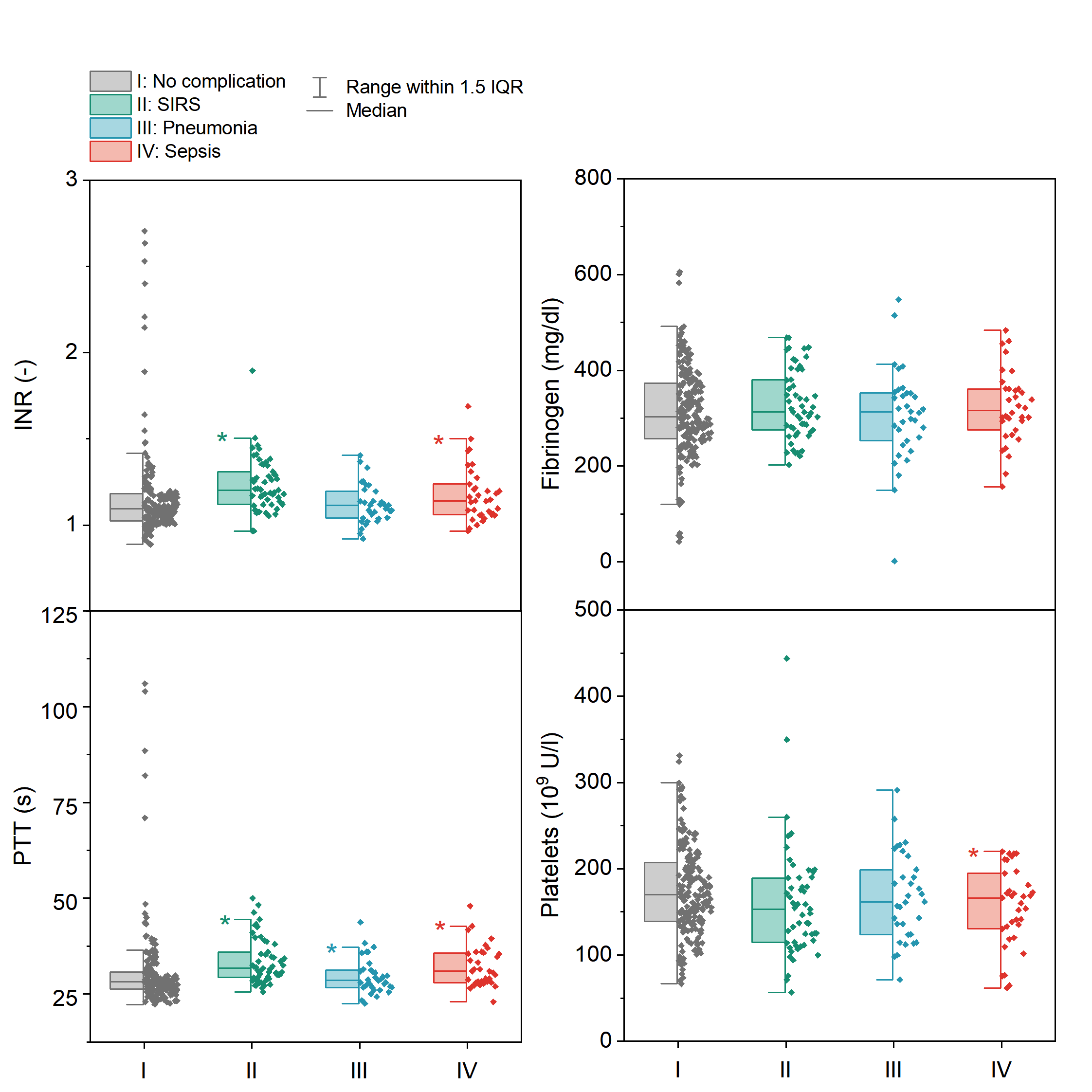

Supplement: Supplementary Figure 4 — Box plots of serum markers for coagulation factors related to the complication risk. Lower and upper box boundaries correspond to 25th and 75th percentiles, respectively. The line inside represents the box median. The lower and upper error lines (whiskers) correspond to the 10th and 90th percentiles, respectively. The asterisk next to the 10th and 90th percentiles indicate statistical significance between the complication group and the patient group having no complications. Points above and below the whiskers indicate outliers outside the 10th and 90th percentiles. Abbreviations: INR, international normalized ratio; PTT, partial thromboplastin time. Supplementary information for statistical tests and correlation analysis [file Image_4.tiff]

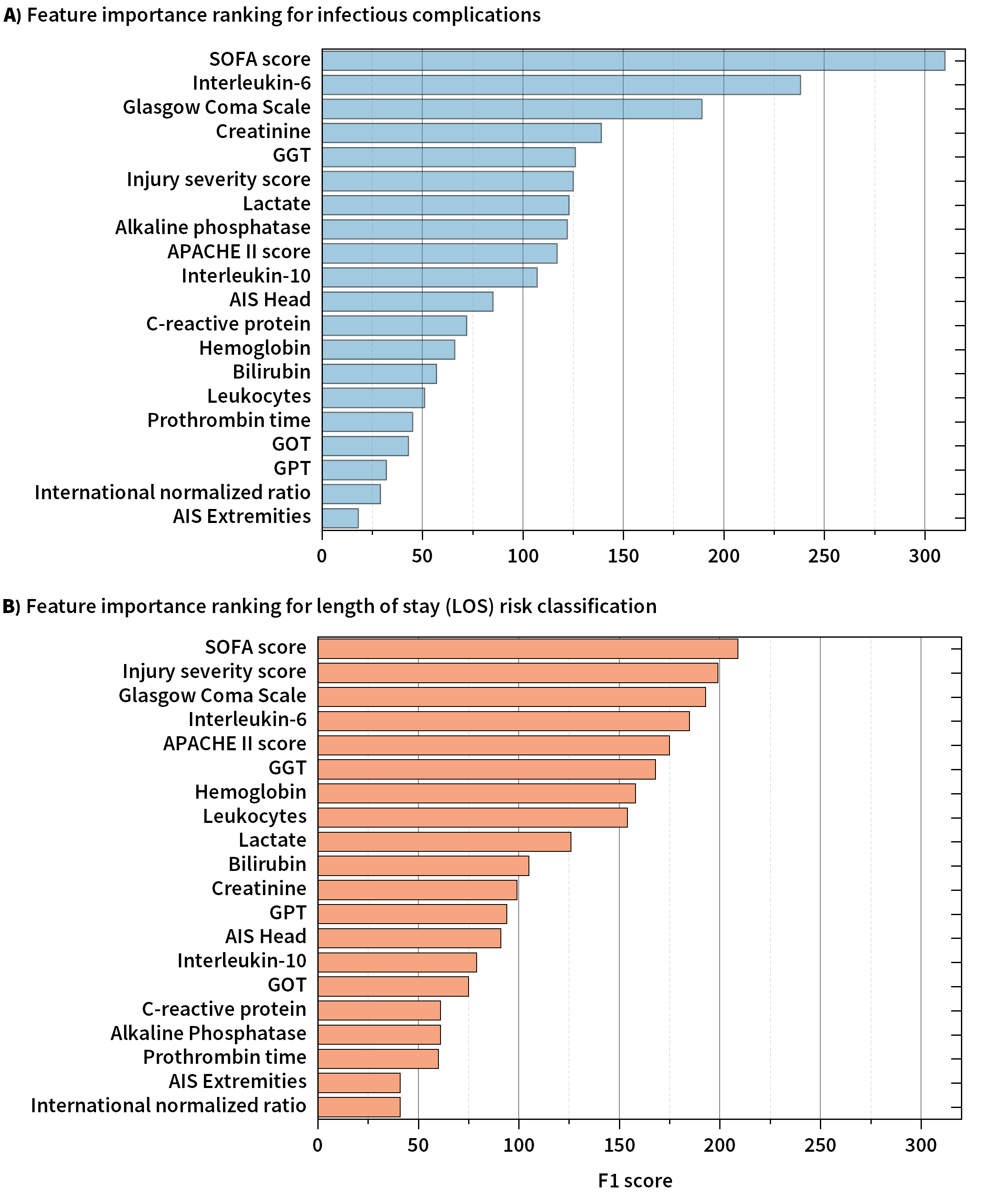

Supplement: Supplementary Figure 5 — Feature importance ranking based on the F1 score with respect to the two investigated risk scenarios: (A) Risk for infectious complications, and (B) risk for a long stay in the intensive care unit (ICU) or total length of stay (LOS) in hospital. Abbreviations: Abbreviations: AIS, abbreviated injury scale; APACHE, acute physiology and chronic health evaluation; GGT, gamma glutamyl transpeptidase; GOT, glutamic oxaloacetic transaminase; GPT, glutamic pyruvic transaminase; SOFA, sequential organ failure assessment. GGT - Gamma glutamyl transpeptidase, GOT-Glutamic oxaloacetic transaminase, GPT-Glutamic pyruvic transaminase. Supplementary information for clustering analysis [file Image_5.tiff]
